# Late Emergence of an Imatinib-Resistant *ABL1* Kinase Domain Mutation in a Patient with Chronic Myeloid Leukemia

**DOI:** 10.1155/2017/3548936

**Published:** 2017-12-11

**Authors:** Mireille Crampe, Claire Andrews, Anne Fortune, Stephen E. Langabeer

**Affiliations:** ^1^Cancer Molecular Diagnostics, St. James's Hospital, Dublin 8, Ireland; ^2^Department of Haematology, Mater Misericordiae University Hospital, Dublin 7, Ireland

## Abstract

The introduction of the tyrosine kinase inhibitor (TKI) imatinib has revolutionised the outlook of chronic myeloid leukemia (CML); however, a significant proportion of patients develop resistance through several mechanisms, of which acquisition of *ABL1* kinase domain mutations is prevalent. In chronic-phase patients, these mutations become evident early in the disease course. A case is described of a chronic-phase CML patient who achieved a sustained, deep molecular response but who developed an Y253H *ABL1* kinase domain mutation nearly nine years after commencing imatinib. Switching therapy to bosutinib resulted in a rapid reachievement of a major molecular response. Long-term TKI treatment impacts on quality of life and late losses of responses are usually due to lack of adherence. This case highlights the requirement for *ABL1* kinase domain mutation analysis in those CML patients on long-term imatinib who lost their molecular response, regardless of whether nonadherence is suspected.

## 1. Introduction

Introduction of the tyrosine kinase inhibitor (TKI) imatinib has revolutionised the treatment of patients with chronic myeloid leukemia (CML) with long-term administration showing persistent efficacy and lack of unacceptable cumulative or late toxic effects [1]. Resistance to imatinib, either primary or acquired, is a recurrent problem in a significant proportion of CML patients that have been largely abrogated by the development and introduction of second- and third-generation TKIs [2]. One of the major causes of imatinib resistance is the development of *BCR-ABL1*-positive clones harboring mutations within the *ABL1* kinase domain (KD) with identification of these mutations as important in selecting a subsequent TKI [3]. Studies have shown that, in newly diagnosed, chronic-phase CML patients, *ABL1* KD mutations predominantly manifest within eighteen months of commencing imatinib and usually in those patients whose best response has only been hematological or cytogenetic [4, 5]. Evidence exists for both increased and low rates of *ABL1* KD mutations in late as opposed to early chronic-phase patients [6, 7]. A CML patient is described in whom an *ABL1* KD mutation was detected nearly nine years after starting imatinib and who had previously achieved a sustained and deep molecular response.

## 2. Case Report

**Figure 1:**
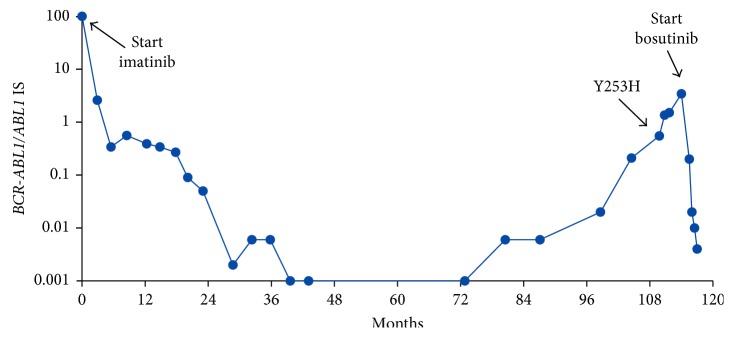
*BCR-ABL1* transcript levels throughout clinical course.

A 49-year-old female presented with nausea, vomiting, and weight loss. Full blood count revealed a white blood cell count of 238.0 × 10^9^/L, hemoglobin of 7.7 g/dL, and platelets of 746 × 10^9^/L. Bone marrow morphology revealed granulocytic hyperplasia with increased megakaryocytes and <1% myeloblasts. Cytogenetics detected the t(9;22) translocation with molecular analysis demonstrating high levels of e13a2 *BCR-ABL1* transcripts, consistent with a diagnosis of chronic-phase CML with a low-risk Sokal score. She commenced imatinib 400 mg oral daily with transient toxicities of nausea and increased susceptibility to infections but overall tolerated imatinib well, achieving a major molecular response (MMR) of *BCR-ABL1/ABL1* 0.09% on the International Scale (IS) at 20 months (Figure 1). *BCR-ABL1* transcripts became undetectable (<0.001% IS) at 40 months with optimal adherence and a good quality of life. After *BCR-ABL1* transcripts became undetectable, monitoring intervals were extended to six months. Rising transcript levels resulted in loss of MMR at 105 months, peaking at a *BCR-ABL1/ABL1* IS of 3.43%, almost nine years after starting imatinib (Figure 1). After adherence was assured, *ABL1* KD mutation analysis was performed as previously described [3, 8] and detected the Y253H (c.757T > C; NM_005157.5) mutation. The time between loss of MMR and mutation detection was six months. The patient then switched to bosutinib 500 mg oral daily [9], reduced to 400 mg oral daily after gastrointestinal toxicities, which resulted in an MMR within three months (*BCR-ABL1*/*ABL1* IS 0.02%). The *BCR-ABL1* transcript level continues to decline (Figure 1) with continued frequent monitoring advocated.

## 3. Discussion

The long-term mild and chronic side effects of TKI therapy may impact on quality of life of CML patients and could be a trigger for lack of adherence [10]. Despite a minor delay in achieving an MMR [11], this patient maintained a sustained molecular response for a significant period of time. *ABL1* kinase domain mutation analysis provided the rationale for switching TKI to bosutinib which induced a rapid molecular response. This case suggests that loss of MMR should always trigger *ABL1* KD mutation analysis even after many years of follow-up, regardless of whether nonadherence is suspected.
